# Atypical Presentation of Interval Colorectal Cancer/Post-Colonoscopy Colorectal Cancer in a Nursing Home Patient

**DOI:** 10.7759/cureus.24849

**Published:** 2022-05-09

**Authors:** Medha R Cherabuddi, Nithin Kurra, Saivishnu Doosetty, Nikhila Gandrakota

**Affiliations:** 1 Internal Medicine, Henry Ford Hospital, Detroit, USA; 2 Neurology, University of Nebraska Medical Center, Omaha, USA; 3 Internal Medicine, Vydehi Institute of Medical Sciences and Research Centre, Bangalore, IND; 4 Family Medicine, Emory University School of Medicine, Atlanta, USA

**Keywords:** deep vein thrombosis, anemia, missed lesion, post-colonoscopy, colorectal cancer

## Abstract

The Centers for Disease Control and Prevention estimates that there are around 1.7 million beds in certified nursing homes across the United States and approximately 1.3 million residents in long-term and end-of-life care. There could be several factors causing a delayed recovery in such patients, such as decreased ambulation, multiple comorbidities, and polypharmacy.

An 83-year-old Caucasian woman sustained a fall resulting in compression fractures of the thoracic and lumbar spine. She had multiple comorbidities, including anemia of chronic disease, malnutrition, and a significant weight loss of 30 lbs over the four months prior to hospitalization. She was on antihypertensives, antidepressants, vitamin D, and calcium supplementation. Her medical history was significant for constipation with the passage of stools once in three days. Her family history was significant for colorectal cancer (CRC) and her screening colonoscopy three years ago was normal. Physical examination revealed no abdominal tenderness or distention. Subsequently, she developed edema in the left lower extremity. She underwent a venous Doppler/ultrasound study, which showed an occlusive thrombus from the common femoral vein to the popliteal vein. She was started on anticoagulants and supportive therapy. Four months later, while at the nursing home, she developed bloating and flatulence, in addition to pre-existing constipation. Examination revealed a 6 x 7 cm mass in the right lower quadrant without peritoneal signs. Bowel sounds were significantly decreased. CT imaging showed a 6-cm diameter cecal mass. The tumor was a low-grade 4 x 9 cm T4N0M0 cecal cancer, and she underwent placement of a Greenfield filter and subsequent hemicolectomy. She had methicillin-resistant *Staphylococcus aureus* infection and right upper extremity deep vein thrombosis (DVT), urinary tract infection, *Clostridium difficile* colitis, and depression, all managed successfully and without sequelae in the post-operative period. Treatment on discharge comprised Coumadin maintenance for nine months with an international normalized ratio goal of 2-3, a back brace, antidepressants, and antihypertensive medications. She received follow-up care at home.

Maintaining a high degree of suspicion for new and persistent symptoms in the elderly is essential to identify the underlying cause. One of the leading causes of post-colonoscopy CRC is a missed lesion. Careful attention to all cases of anemia as well as DVT in the elderly is also imperative to diagnose such missed cases. Future research should focus on the methods of CRC diagnosis in elderly patients with comorbidities apart from using colonoscopy alone.

## Introduction

The Centers for Disease Control and Prevention (CDC) estimates around 1.7 million beds in certified nursing homes across the United States and approximately 1.3 million residents in long-term and end-of-life care [1]. Most patients undergo skilled rehabilitation before they go home. There could be several factors causing a delayed recovery in such patients. Inadequate pain control, multiple comorbidities, polypharmacy, infections, delirium, dementia, depression, and undernutrition are a few factors. Decreased mobility due to the lack of participation aggravated our patient’s risk of thromboembolism. We noticed a right lower quadrant (RLQ) mass that raised the suspicion of malignancy. The patient had no prior history of abnormal colonoscopies, including her most recent routine colonoscopy three years ago. A CT scan identified the mass, and a subsequent laparotomy confirmed the colon’s cancer diagnosis.

## Case presentation

An 83-year-old Caucasian woman sustained a fall resulting in compression fractures of the thoracic and lumbar spine. Due to severe pain, she underwent kyphoplasty of the third lumbar vertebra and first thoracic vertebra. Inadequate pain control prolonged her post-operative stay of four weeks. She had multiple comorbidities, including anemia of chronic disease, malnutrition, and a significant weight loss of 30 lbs four months before hospitalization. She was on antihypertensives, antidepressants, vitamin D, and calcium supplementation. Her medical history was significant for constipation with the passage of stools once in three days, which was assumed to be a side effect of her pain medication, tramadol. The management included increased fluid intake and prune juice. Her family history was significant for colorectal cancer (CRC), and her screening colonoscopy three years ago was regular. Physical examination revealed no abdominal tenderness or enlargement. Subsequently, she developed edema in the left lower extremity. She underwent a venous Doppler/ultrasound study, which showed an occlusive thrombus from the common femoral vein to the popliteal vein, likely due to immobility during this period. She was started on anticoagulants and supportive therapy. Four months later, while at the nursing home, she developed bloating, flatulence, and pre-existing constipation. Examination revealed a 6 x 7 cm mass in the RLQ without peritoneal signs. Bowel sounds were significantly decreased. The patient refused a rectal examination. CT imaging showed a 6-cm diameter cecal mass, which led to the suspicion of malignancy. The tumor was a low-grade 4 x 9 cm T4N0M0 cecal cancer, and she underwent placement of a Greenfield filter and subsequent hemicolectomy. The patient refused chemotherapy. She had methicillin-resistant *Staphylococcus aureus* infection and right upper extremity deep vein thrombosis (DVT), urinary tract infection, *Clostridium difficile* colitis, and depression, all managed successfully and without sequelae in the post-operative period. Treatment on discharge comprised Coumadin maintenance for nine months with an international normalized ratio goal of 2-3, a back brace, antidepressants, and antihypertensive medications. She received follow-up care at home.

## Discussion

According to the American Cancer Society, there will be a projected 106,180 new colon cancer cases and 44,850 cases of rectal cancer in the United States in 2022, with an estimated 52,580 deaths due to this cancer [2]. In comparison to the 2018 data from the CDC, 141,074 cases of colon and rectum cancer were reported, with 52,163 deaths [3]. The majority of CRC cases (approximately 88%) occur in patients aged 50 years or older [4].

The United States Preventive Services Task Force (USPSTF) recommends screening to be performed between 50 and 75 years of age, while adults aged 76-85 may benefit if they have never been screened before and are in good health with few co-morbidities to tolerate treatment in the case of colorectal cancer detected [5]. Screening is not recommended beyond 85 years of age. Fecal immuno-chemical tests (FITs) are more sensitive than the fecal occult blood test (FOBT), and flexible sigmoidoscopy, when combined with the FIT, was found to reduce the CRC-specific mortality rate more than flexible sigmoidoscopy alone [5]. The most common methods of CRC screening are described in Table 1.

**Table 1:** Screening methods for colorectal cancer y, years; RCT, randomized control trial; FIT, fecal immuno-chemical test; gFOBT, guaiac fecal occult blood test

Screening method	Frequency	Evidence of efficacy	Other considerations
Stool-based tests			
gFOBT	Every year	RCTs with mortality endpoints: high-sensitivity versions (e.g., Hemoccult Sensa) have superior test performance characteristics than older tests (e.g., Hemoccult II)	Does not require bowel preparation, anesthesia, or transportation to and from the screening examination (test at home)
FIT	Every year	Test characteristic studies: improved accuracy compared with gFOBT, can be done with a single specimen	Does not require bowel preparation, anesthesia, or transportation to and from the screening examination (test at home)
FIT-DNA	Every 1 or 3 y	Test characteristic studies: specificity is lower than FIT, resulting in more false-positive results, more diagnostic colonoscopies, and more associated adverse events per screening test; improved sensitivity compared with FIT per single screening test	There is insufficient evidence about the appropriate longitudinal follow-up of abnormal findings after a negative diagnostic colonoscopy, which may lead to overly intensive surveillance due to provider and patient concerns over the genetic component of the test
Direct visualization tests			
Colonoscopy	Every 10 y	Prospective cohort study with mortality endpoint	Requires less frequent screening; screening and diagnostic follow-up of positive results during the same examination
CT colonography	Every 5 y	Test characteristic studies	There is insufficient evidence about the potential harms of associated extracolonic findings, which are common
Flexible sigmoidoscopy	Every 5 y	RCTs with mortality endpoints: modeling suggests it provides less benefit than when combined with FIT or compared with other strategies	Test availability has declined in the United States
Flexible sigmoidoscopy with FIT	Flexible sigmoidoscopy every 10 y plus FIT every year	RCT with mortality endpoint (subgroup analysis)	Test availability has declined in the United States; potentially attractive option for patients who want endoscopic screening but want to limit exposure to colonoscopy

Colonoscopy screening at the age of 80 turned out negative in our patient. The symptoms of colorectal cancer vary based on the site of the lesion. A summary of the varied symptoms of CRC is presented in Table 2.

**Table 2:** Symptoms of colorectal cancer

Local	Systemic
Changes in the bowel habit	Unintentional weight loss
Constipation	Anorexia, nausea, or vomiting
Diarrhea	Fatigue
Alternating diarrhea and constipation	Anemia
Bright red stools	Jaundice
Tarry stools	
Abdominal discomfort, bloating, and cramping	

In this patient, along with anemia, weight loss, and constipation, the disease was locally advanced on presentation, despite regular screening. Therefore, we must pay close attention to the above-mentioned symptoms in addition to screening, especially in the elderly. Robertson et al. estimated that 52% of interval cancers were likely due to missed lesions, 24% due to possible new lesions and 19% due to incompletely excised lesions [6]. A population-based study in Canada also found that colonic cancers were more likely to be missed in women than in men for unclear reasons, and the more proximal the lesion, the more likely it is to be ignored [7]. Our patient also most likely falls in this category of a missed proximal lesion. Another study showed that 20% of the overall interval colorectal cancers (ICRCs) were mismatch repair (MMR) deficient by immunohistochemistry, and this subset showed a female predominance [8]. The proximal location is most likely related to the higher prevalence of flat precursor lesions in the right colon, which may be more challenging to detect and excise entirely than their more polypoid and pedunculated counterparts in the left colon [8]. In a population-based cross-sectional study of incident CRC cases in Utah, post-colonoscopy CRCs were more likely to arise proximally and demonstrate microsatellite instability with tumorigenesis features or processes [9].

Our patient had a history of anemia, and DVT warrants close attention, especially in the elderly, as seen in our patient. The importance of lower gastrointestinal evaluation in iron-deficiency anemia in people over 50 years of age is well documented [10]. Unexplained iron-deficiency anemia, with a substantial decrease in hemoglobin, mean corpuscular volume, and serum ferritin, is an important marker for CRC diagnosis [11]. Of particular importance is that CRC is a common cause of ambulatory diagnostic malpractice claims in the United States. The most common reason was a failure to perform a warranted test [12]. Evidence shows that anemia is the most common clue associated with the longest time to endoscopy referrals [13].

The next clue indicating occult cancer was the presence of right upper extremity DVT. DVT is expected in this age group, especially after spinal surgery and poor ambulation in women. Another study shows a high prevalence of preoperative DVT in patients in need of CRC surgery, and the increasing incidence of venous thromboembolism (VTE) in all cancers is underestimated [14]. Clotting pathway factors, including the tissue factor, might be helpful as biomarkers in CRC for the assessment of VTE risk and cancer prognosis. VTE in CRC is challenging to manage and is a significant mortality predictor within one year of the diagnosis, even with a promising forecast suggesting an association with biologically aggressive cancers [15].

Prior evidence shows an inverse relationship between the incidence and severity of colonic cancer and calcium and vitamin D levels [16]. Our patient had a history of vitamin D and calcium deficiency.

One study encourages endoscopists to include the risk of ICRC in the informed consent process since missed or incompletely resected lesions are more likely than new aggressive biology. Multiple methods to improve colonoscopy quality have also been proposed [17]. This might positively affect the course of management in patients with missed lesions.

## Conclusions

Maintaining a high degree of suspicion for new and persistent symptoms in the elderly is essential to identify the underlying cause of CRC. One of the leading causes of post-colonoscopy CRC is a missed lesion, which is more likely if the patient is female, and the lesion is proximal in location. Hence, endoscopists should consider the risk of missed lesions, especially in patients with clinical symptoms aligning with the risk of CRC. Careful attention to all cases of anemia and DVT in the elderly is also imperative to diagnose such missed issues. Future research should focus on the methods of CRC diagnosis in elderly patients with comorbidities apart from using colonoscopy alone.

## References

[1] (2021). Nursing home care. https://www.cdc.gov/nchs/fastats/nursing-home-care.htm.

[2] (2020). Key statistics for colorectal cancer. American Cancer Society.

[3] (2021). U.S. Cancer Statistics data visualizations, based on 2020 submission data (1999-2018). https://gis.cdc.gov/Cancer/USCS/DataViz.html.

[4] American Cancer Society (2020). Colorectal Cancer Facts & Figures 2020-2022. American Cancer Society.

[5] (2021). United States Preventive Services Task Force: Final recommendation statement. Colorectal cancer: screening. https://www.uspreventiveservicestaskforce.org/uspstf/recommendation/colorectal-cancer-screening.

[6] Robertson DJ, Lieberman DA, Winawer SJ (2014). Colorectal cancers soon after colonoscopy: a pooled multicohort analysis. Gut.

[7] Singh H, Nugent Z, Demers AA, Bernstein CN (2010). Rate and predictors of early/missed colorectal cancers after colonoscopy in Manitoba: a population-based study. Am J Gastroenterol.

[8] Soong TR, Nayor J, Stachler MD (2019). Clinicopathologic and genetic characteristics of interval colorectal carcinomas favor origin from missed or incompletely excised precursors. Mod Pathol.

[9] Samadder NJ, Neklason D, Snow A (2019). Clinical and molecular features of post-colonoscopy colorectal cancers. Clin Gastroenterol Hepatol.

[10] Goddard AF, James MW, McIntyre AS, Scott BB (2011). Guidelines for the management of iron deficiency anaemia. Gut.

[11] Schneider C, Bodmer M, Jick SS, Meier CR (2018). Colorectal cancer and markers of anemia. Eur J Cancer Prev.

[12] Aaronson EL, Quinn GR, Wong CI, Murray AM, Petty CR, Einbinder J, Schiff GD (2019). Missed diagnosis of cancer in primary care: Insights from malpractice claims data. J Healthc Risk Manag.

[13] Wahls TL, Peleg I (2009). Patient- and system-related barriers for the earlier diagnosis of colorectal cancer. BMC Fam Pract.

[14] Nakagawa K, Watanabe J, Suwa Y (2019). Clinical analysis of preoperative deep vein thrombosis risk factors in patients with colorectal cancer: retrospective observational study. Ann Gastroenterol Surg.

[15] Rees PA, Clouston HW, Duff S, Kirwan CC (2018). Colorectal cancer and thrombosis. Int J Colorectal Dis.

[16] Welsh J (2012). Cellular and molecular effects of vitamin D on carcinogenesis. Arch Biochem Biophys.

[17] Patel SG, Ahnen DJ (2014). Prevention of interval colorectal cancers: what every clinician needs to know. Clin Gastroenterol Hepatol.

